# P-1458. Awareness, Attitudes and Perceptions of Meningococcal Vaccines among Caregivers of Adolescents in the United States

**DOI:** 10.1093/ofid/ofaf695.1644

**Published:** 2026-01-11

**Authors:** Jessica Presa, Daniel Spitz, Ronika Alexander, Soohyun Hwang, Gabriela Burgos, Grace Skiles, Paul Palmer

**Affiliations:** Pfizer, Inc., Collegeville, PA; Pfizer Inc, Collegeville, Pennsylvania; Pfizer, Washington, District of Columbia; Oracle Life Sciences, Santa Clara, California; Oracle Life Sciences, Santa Clara, California; Oracle, Redding, California; Pfizer Vaccine Medical Development, Scientific & Clinical Affairs , Collegeville PA, Collegeville, PA

## Abstract

**Background:**

Meningococcal disease (MD) is most commonly observed in infants, with a second incidence peak occurring in adolescents and young adults in the United States. The Centers for Disease Control and Prevention recommends vaccination among preteens, teenagers, and young adults; however, adolescent vaccination rates are suboptimal. This study aimed to gain insights on awareness, attitudes, and perceptions of vaccines, particularly those for MD, among caregivers (CGs) of adolescents.

**Figure:**
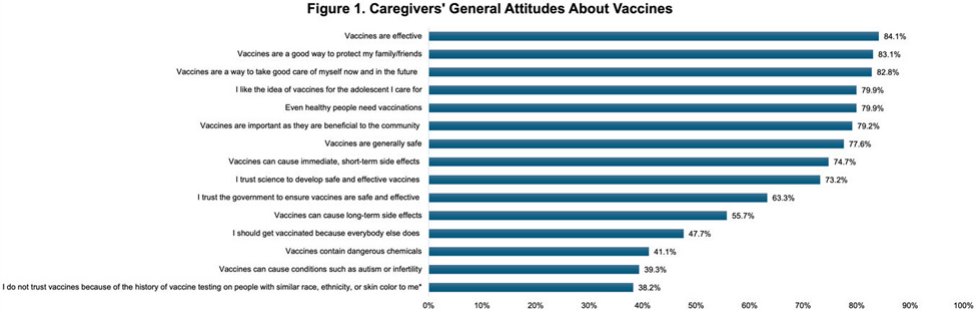
Caregivers' General Attitudes About Vaccines

**Methods:**

A cross-sectional online survey was conducted with US adults who are the primary or co-primary CG of an adolescent aged 16-18 years. Data on awareness and experience with MD and vaccines, attitudes and perceptions of vaccines, and demographics were collected. Descriptive statistics were used to summarize the results.

**Results:**

Of 384 CGs who were included in analyses, 52.9% were female (mean age: 45.6 years), and 20.8% were Hispanic/Latino. Among those CGs whose adolescent received a MD vaccine (n=202), the most influential reason for their decision was healthcare provider (HCP) recommendation (80.7%), followed by an HCP providing sufficient information for them to feel comfortable about the vaccine (41.6%). The Meningococcal Serogroup ACWY (MenACWY 41.6%) and Meningococcal Serogroup B (MenB; 24.3%) vaccines were most often received. Of those whose HCP recommended the MenB vaccine (n=69), CGs were most often advised to wait until the adolescent was 16 years old (55.1%). Most CGs slightly/strongly agreed that vaccines, in general, are effective (84.1%) and safe (77.6%); yet, 39.3-55.7% slightly/strongly agreed with the statements that vaccines can cause long-term side effects, cause autism or infertility, or contain dangerous chemicals (Figure 1).

**Conclusion:**

CGs who had their adolescent vaccinated against MD were most influenced by HCP recommendations. However, many CGs held erroneous beliefs about the safety of vaccines, in general. These findings highlight an opportunity for HCPs to better inform CGs about the vaccines they recommend for adolescents, including those for MD. Potential subgroup differences (e.g., race/ethnicity) in CG attitudes and perceptions are currently being explored.

**Disclosures:**

Jessica Presa, MD, Pfizer Inc: Industry|Pfizer Inc: Stocks/Bonds (Public Company) Daniel Spitz, PharmD, Pfizer Vaccines: Employee|Pfizer Vaccines: Stocks/Bonds (Public Company) Ronika Alexander, M.Ed, Pfizer, Inc.: employee|Pfizer, Inc.: Stocks/Bonds (Private Company) Soohyun Hwang, PhD, MPH, Pfizer: Employed by Oracle Life Sciences, which received funding from Pfizer to conduct the study. Grace Skiles, MA, Pfizer: Employed by Oracle Life Sciences, which received funding from Pfizer to conduct the study Paul Palmer, PhD, Pfizer Inc: Employee|Pfizer Inc: Stocks/Bonds (Public Company)

